# The influence of urban versus rural residence and travel distance on net income, out-of-pocket payments and quality of life in German head and neck cancer survivors

**DOI:** 10.1007/s00432-026-06534-5

**Published:** 2026-07-18

**Authors:** Jonas Rast, Theresa Wald, Veit Zebralla, Andreas Dietz, Gunnar Wichmann, Susanne Wiegand

**Affiliations:** 1 Department of Otorhinolaryngology, Head and Neck Surgery; Phoniatrics and Pediatric Audiology, University Hospital Schleswig-Holstein, Campus Kiel, Arnold-Heller-Str. 3, 24105 Kiel, Germany; 2 Department of Otorhinolaryngology, Head and Neck Surgery, University Hospital Leipzig, Liebigstr. 10-14, 04103 Leipzig, Germany

**Keywords:** Head and neck cancer, Financial burden, Distance, Out-of-pocket payment (OOPP), Travel cost, Quality of life (QoL)

## Abstract

**Purpose:**

German HNC survivors frequently experience substantial financial burden (FB). Geographic distance to cancer care centers (CCC) may increase FB negatively affecting QoL and outcome by creating barriers to diagnosis, treatment, and aftercare. We evaluated the impact of distance to CCC on FB and provide insights into socioeconomic conditions of HNC patients.

**Methods:**

Between August 2022 and March 2023, 200 HNC patients completed a comprehensive questionnaire assessing financial aspects, including self-reported FB, and the EORTC QLQ-C30. Distances from each patient’s residence to the treating CCC were recorded and analyzed in relation to socioeconomic characteristics. Parametric and non-parametric statistical methods were applied to assess subgroup differences associated with travel distance.

**Results:**

The mean travel distance was 27.9 km (95% CI 20.3–35.5). Patients residing in rural areas and farther from aftercare centers reported significantly higher out-of-pocket payments due to travel expenses and additional costs. A significant income disparity was observed between HNC patients living in urban cores and the general population within the same ZIP code (Δ − 499.56€; *p* = 0.014). Rural patients reported significantly higher financial difficulty scores on the EORTC QLQ-C30, reflecting greater FB and impaired QoL (*p* < 0.001). Overall, HNC survivors had significantly lower income than the general population, with the lowest levels observed among urban patients.

**Conclusion:**

Increased distance to CCC is associated with higher FB and reduced QoL among HNC survivors, even in a statutory healthcare system. These disparities highlight the need for targeted interventions to reduce financial toxicity and improve access to cancer aftercare.

**Supplementary Information:**

The online version contains supplementary material available at 10.1007/s00432-026-06534-5.

## Introduction

Cancer patients have to face numerous obstacles before they can access tailored diagnostics and treatment (Douthit et al. 2015). Previous studies have documented a variety of challenges, including psychological, economic, social, and sanitary issues (Ambroggi et al. 2015). The economic aspect of cancer care, particularly for survivors, is increasingly recognized as a vital part of patients' well-being, and has become a major area of research (Khan et al. 2025). Recent research has demonstrated that financial burden is a significant factor in cancer survivorship, influencing quality of life even in patients with similar cancer characteristics (Rast et al. 2024).

In addition, as cancer patients require multiple visits to physicians or hospitals, travel distance is an important factor for them to consider during their treatment (Guidry et al. 1997). Indeed, time costs associated with travel represent a significant component of the overall economic burden of cancer.

In light of the aforementioned factors, it is evident that cancer survivors, particularly those patients with head and neck cancer (HNC), may be disproportionately affected, with some experiencing greater disadvantages than others (Massa et al. 2019; Zebralla et al. 2018; Wu et al. 2022) Evidence is emerging that even countries with statutory health insurance are facing these challenges (Mehlis et al. 2020).

Rural cancer patients are confronted with considerable transportation costs, as they are required to travel longer distances to access the same level of clinical care as their urban counterparts. Furthermore, they often lack access to public or private transportation, further exacerbating this issue (Petermann et al. 2022). For socioeconomically disadvantaged patients, travel can be of particular importance, given the significant time costs associated with care which can strain limited resources.

An increasing number of studies have defined this phenomenon as travel burden, assessed by distance or time, which may lead to delays in diagnosis and affect treatment decision-making across various common cancers (Ambroggi et al. 2015; Lee et al. 2014; Birkmeyer et al. 2003; Stitzenberg et al. 2007).

On the other hand, accumulated evidence suggests that cancer patients are treated more efficiently at high-volume centers and especially certified oncological care centers than at low-volume centers (Freeman et al. 2010). Therefore, it is vital that cancer patients are treated at such centers in order to achieve the best possible clinical outcomes.

As centralization of care increases, the number of hospitals providing cancer aftercare is decreasing. Consequently, more patients may need to travel longer distances to receive specialized care.

Less is known about the living conditions of survivors of HNC in Germany and their travel distance to cancer care programs. With regard to the location of the patient, it would be beneficial to ascertain specific information about their needs in order to facilitate future research aimed at mitigating financial burden and enhancing their quality of life.

To evaluate the potential influence of financial and travel-related burdens on patient outcomes, we analyzed a large group of patients from a HNC center situated in eastern Germany.

## Methods

### Study design and participants

The present study was approved by the Ethics Committee of the Medical Faculty of the University of Leipzig (vote 289,722-ek) and conducted in accordance with the ethical standards of the 1964 Declaration of Helsinki and its subsequent amendments. German-speaking adult patients with head and neck squamous cell carcinoma (HNSCC) treated at the Department of Otorhinolaryngology, Head and Neck Surgery of the University of Leipzig and enrolled in the aftercare program between August 2022 and March 2023 were eligible for participation. Inclusion criteria required participants to be at least 18 years of age. Patients were invited consecutively during follow-up consultations, typically two to three months after completion of active HNSCC treatment. A total of 209 patients provided written informed consent.

### Survey instrument

Clinical and disease-related data, including sex, age, tumor site and stage, recurrence, and treatment regimen, were extracted from patient records. Patient travel distance (km) by car was calculated by using their postal code and the address of the treating university hospital. For calculation Google maps (accessed between April-July 2024) and for geographical mapping Microsoft Excel was used. Data on monthly mean net income per zip code were obtained from the 2021 results of the Micro census (largest annual household survey of official statistics in Germany) from Saxony (https://www.statistik.sachsen.de/html/statistische-aemter-bund-laender.html).

Information on financial burden (FB) was derived from a previously published analysis of the same cohort (Rast et al. 2024), based on a self-administered questionnaire assessing out-of-pocket payments (OOPP; non-reimbursed additional costs), household income and income loss, employment status, and social consequences. An English version of the questionnaire is available in the supplementary material (Questionnaire 1). All participating patients had sufficient proficiency in German to independently complete the questionnaire, with on-site assistance available to address any questions that arose. The survey was completed only once by each patient.

Quality of Life (QoL) and related behavioral outcomes were assessed using the European Organization of Research and Treatment of Cancer Quality of Life Questionnaire Core 30 (EORTC QLQ-C30) (Aaronson et al. 1993). The instrument includes five functional domains (physical, role, cognitive, emotional, and social functioning), three symptom scales (fatigue, pain, nausea and vomiting), and a global health status/quality-of-life scale. Additional single items assess common cancer-related symptoms, including dyspnoea, appetite loss, sleep disturbance, constipation, and diarrhoea, as well as financial difficulties. Scoring and interpretation followed the validated EORTC guidelines as described by Aaronson et al. (1993).

### Statistical analysis

Parametric and non-parametric statistics were used to elucidate differences in FB among patient subgroups attributable to the travel distance. Receiver operating characteristic (ROC) curves were used to assess the predictive value of travel distance for OOPP and to calculate sensitivity, specificity and *Youden’s J* (*J* = sensitivity + specificity − 1) as measure to detect local maxima providing useful thresholds to define cut-off values for classification. The distribution of data among groups was compared using chi-squared (χ^2^) tests for categorical data, as well as non-parametric *Mann–Whitney U* or *Kruskal–Wallis* tests with *Bonferroni* correction for multiple testing, as appropriate. In addition to inferential statistics, effect sizes were calculated using *Cohen´s d* to quantify the magnitude of observed effects.

### Multivariable analyses

Multivariable analyses were done applying logistic regression. For these analyses, clearly defined information from EORTC QoL Q30 for financial difficulties (FD Scale 0 vs. 1 for any reported difficulty) and the median Global QoL Score of the sample were used as outcome. Three independent risk factors for financial burden elsewhere reported (Rast et al. 2024, 2025) were included as covariates ((1) advanced stage cancer, (2) T3 or T4 category of the primary tumor, (3) localization of the primary tumor in either larynx or hypopharynx) to analyze the association of urban versus rural residence and traveled distance with OOPP and QoL.

## Results

The analyzed cohort consisted of 200 patients with complete questionnaires. The mean age was 64.2 years, with 72.7% of the participants being male. The majority of the participants had been diagnosed with an advanced UICC (III or IV) stage (*n* = 137). Table 1 presents demographics, employment status and tumor-related characteristics, including site, histology, classification, and treatment, stratified by distance groups (“Leipzig”, “outside of Leipzig < 28.1 km” and “outside of Leipzig > 28.1 km”) and related OOPP. The ROC for travel distance for OOPP had an AUC of 0.694 (95% CI 0.59–0.798; *p* = 0.00081) and revealed two local maxima, the first was identical to Leipzig city area (travel distance up to 10 km, sensitivity 59.5%, specificity 78.9%, *Youden's J* 0.384) and the second was at 28.1 km (sensitivity 77.0%, specificity 57.9%, *Youden's J* 0.349).

**Table 1:** Cancer and social-demographic characteristics of the cohort

Variables	Total (valid values) No. (%)	Leipzig (*n* = 111) No. (%)	Outside Leipzig < 28.1 km (*n* = 37) No. (%)	Outside Leipzig > 28.1 km (*n* = 61) No. (%)	*p*-Value
Age score					0.5509
18–50 years	14 (6.7)	9 (4.3)	0 (0)	5 (2.4)	
51–60 years	50 (23.9)	27 (12.9)	7 (3.3)	16 (7.7)	
61–70 years	89 (42.6)	44 (21.1)	19 (9.1)	26 (12.4)	
> 70 years	56 (26.8)	31 (14.8)	11 (5.3)	14 (6.7)	
Sex					0.5784
Male	152 (72.7)	80 (38.3)	25 (12.0)	47 (22.5)	
Female	57 (27.3)	31 (14.8)	12 (5.7)	14 86.7)	
p16 OPSCC versus other					0.1542
p16 + OPSCC	58 (27.8)	27 (12.9)	15 (7.2)	16 (7.7)	
Other	151 (72.2)	84 (40.2)	22 (10.5)	45 (21.5)	
OP (monomodal)					0.0417
Yes	51 (24.4)	34 (16.3)	4 (1.9)	13 (6.2)	
No	158 (75.6)	(36.8)	33 (15.8)	48 (23.0)	
OP_ADJ					0.1199
Yes	115 (55.0)	57 (27.3)	26 (12.4)	32 (15.3)	
No	94 (45.0)	54 (25.8)	11 (5.3)	29 (13.9)	
PRIM_RT_RCHT					0.1313
Yes	35 (16.7)	14 (6.7)	6 (2.9)	15 (7.2)	
No	174 (83.3)	97 (46.4)	31 (14.8)	46 (22.0)	
N3 versus other					0.4214
N3 7th ed	7 (3.3)	5 (2.4)	0 (0)	2 (1.0)	
Other	189 (90.4)	100 (47.8)	35 (16.7)	54 (25.8)	
Invalid/unkown	13 (6.2)				
T4 versus other					0.0704
T4 7th ed	31 (14.8)	13 (6.2)	10 (4.8)	8 (3.8)	
Other	165 (78.9)	92 (44.0)	25 (12.0)	48 (23.0)	
Invalid/unkown	13 (6.2)				
T3, T4 versus other					0.1285
T3 or T4 7th ed	77 (36.8)	38 (18.2)	19 (9.1)	20 (9.6)	
Other	132 (63.2)	73 (34.9)	18 (8.6)	41 (19.6)	
ENE					0.1287
ENE	44 (21.1)	24 (11.5)	12 (5.7)	8 (3.8)	
no ENE	63 (30.1)	37 (17.7)	8 (3.8)	18 (8.6)	
N/A. N0	80 (38.3)	41 (19.6)	11 (5.3)	28 (13.4)	
Invalid/unkown	22 (10.5)				
Larynx/hypopharynx versus other					0.5442
Larynx/hypopharynx	53 (25.4)	31 (14.8)	7 (3.3)	15 (7.2)	
Other localization	156 (74.6)	80 (38.3)	30 (14.4)	46 (22.0)	
Advanced versus early					0.0792
Advanced	137 (65.6)	70 (33.5)	30 (14.4)	37 (17.7)	
Early	59 (28.2)	35 (16.7)	5 (2.4)	19 (9.1)	
Invalid/unkown	13 (6.2)				
Employment status at time of diagnosis					0.5287
Employment	88 (42.1)	45 (21.5)	16 (7.7)	27 (12.9)	
Self-employment	9 (4.3)	4 (1.9)	2 (1.0)	3 (1.4)	
Civil servant	2 (1.0)	0 (0)	0 (0)	2 (1.0)	
Part-time employment	7 (3.3)	4 (1.9)	0 (0)	3 (1.4)	
No employment	29 (13.9)	16 (7.7)	7 (3.3)	6 (2.9)	
Retired	70 (33.5)	40 (19.1)	10 (4.8)	20 (9.6)	
Invalid/unkown	4 (1.9)				
Net income categories per month before tumor diagnosis					0.1718
No own income	8 (3.8)	5 (2.4)	2 (1.0)	1 (0.5)	
1–500€	9 (4.3)	3 (1.4)	0 (0)	6 (2.9)	
501–1000€	17 (8.1)	10 (4.8)	4 (1.9)	3 (1.4)	
1001–1500€	27 (12.9)	17 (8.1)	2 (1.0)	8 (3.8)	
1501–2000€	22 (10.5)	10 (4.8)	6 (2.9)	6 (2.9)	
2001–2500€	20 (9.6)	10 (4.8)	2 (1.0)	8 (3.8)	
2501–3000€	10 (4.8)	4 (1.9)	4 (1.9)	2 (1.0)	
3001–3500€	3 (1.4)	1 (0.5)	1 (0.5)	1 (0.5)	
> than 3500€	7 (3.3)	1 (0.5)	3 (1.4)	3 (1.4)	
Retired	70 (33.5)	40 (19.1)	10 (4.8)	20 (9.6)	
Invalid/unkown	16 (7.7)				
Current employement status					0.7313
Employment	45 (21.5)	23 (11.0)	7 (3.3)	15 (7.2)	
Self-employment	5 (2.4)	3 (1.4)	1 (0.5)	1 (0.5)	
Civil servant	1 (0.5)	0 (0)	0 (0)	1 (0.5)	
Part-time employment	4 (1.9)	3 (1.4)	0 (0)	1 (0.5)	
No employment	22 (10.5)	14 (6.7)	3 (1.4)	5 (2.4)	
Retired after cancer diagnosis	54 (25.8)	24 (11.5)	14 (6.7)	16 (7.7)	
Retired before cancer diagnosis	70 (33.5)	40 (19.1)	10 (4.8)	20 (9.6)	
Invalid/unkown	8 (3.8)				

Stratification by distance showed no significant differences in terms of sex, age or employment status. The proportion of patients in the 61–70 and > 70 age groups was higher in the Leipzig subgroup, though this did not reach statistical significance. The employment rate was higher outside of Leipzig than inside, but this difference was also not statistically significant. There were significantly more patients who were treated with surgery alone in Leipzig compared to the two subgroups outside Leipzig (*n* = 34, *p* = 0.0417). There were no statistically significant differences in the prevalence of p16-positive OPSCC among the subgroups.

**Fig. 1:**
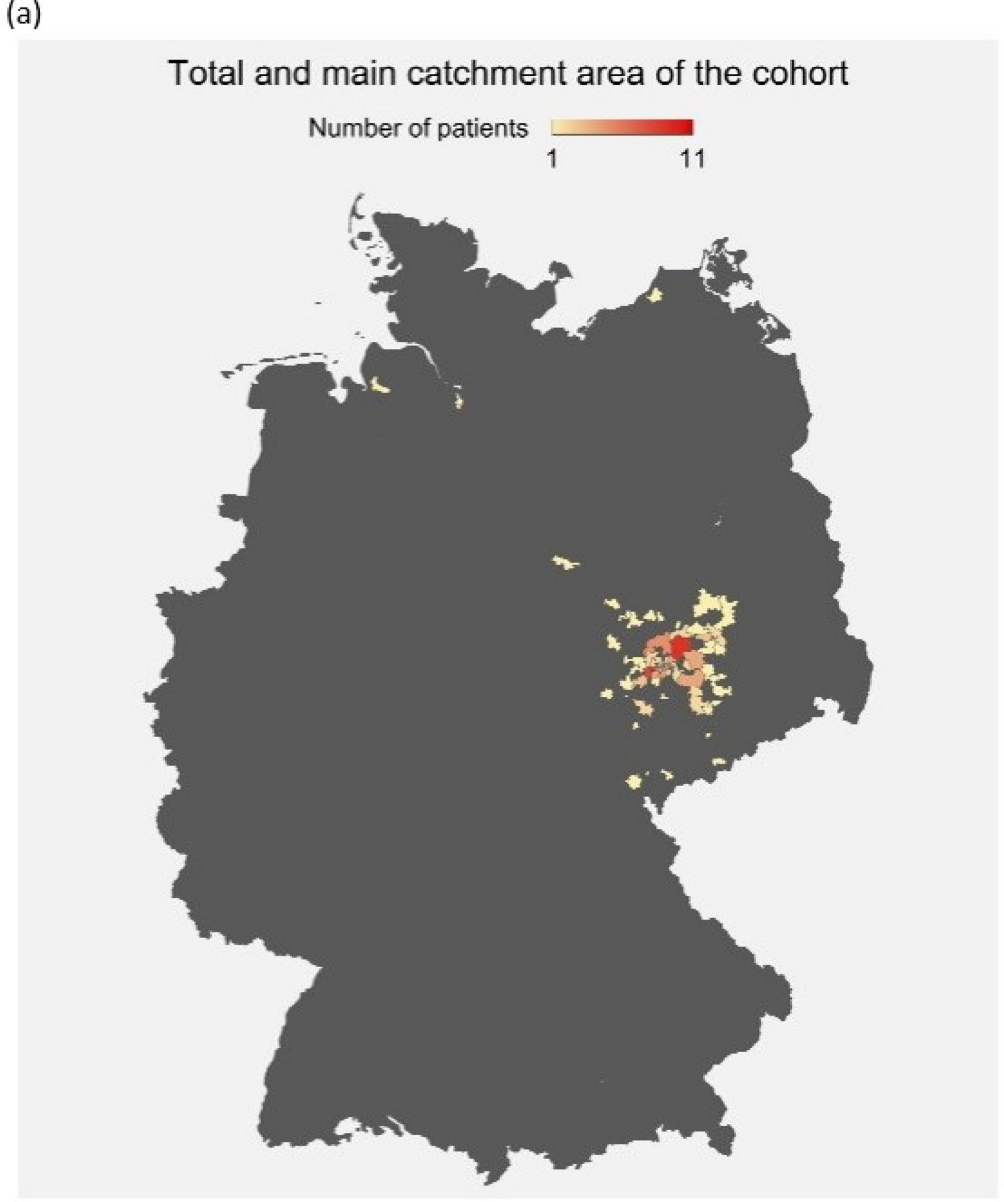

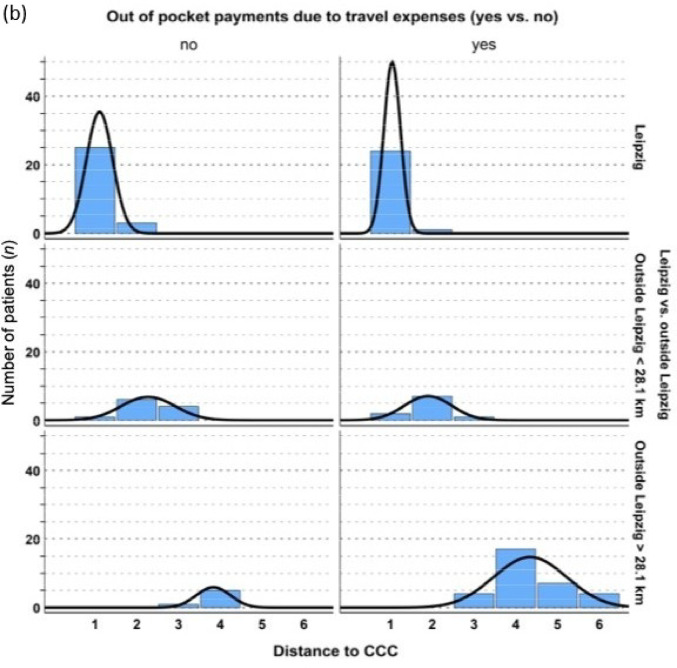
**a** Geographical distribution of the participating HNC survivors in a catchment chart by using their domestic postal codes. **b** Distribution in number of patients in Leipzig (upper row), outside Leipzig within 28.1 km (middle row) and beyond 28.1 km to the cancer care center (CCC) without (no; left panel) and with increased out of pocket payments due to travel expenses (yes; right panel) according to distance to the CCC: 1 = 50th, 2 = 66th, 3 = 75th, 4 = 90th, 5 = 95th percentile, and 6 = above

Figure 1a portrays the geographical distribution of the participating HNC survivors in a catchment chart by using their domestic postal codes. The main catchment area is within the state of Saxony and the city of Leipzig. The mean travel distance for the entire cohort was 27.9km (95% CI 20.3–35.5km). Figure 1b shows the relation between travel distance (in km) to the University Hospital Leipzig and OOPP and the heightened number of patients living ≥ 28.1 km away from the CCC.

A sub-analysis comparing travel distance to the CCC between patients with a pension and those without demonstrated that patients with a pension lived significantly (*p* = 0.033) closer to the CCC. The mean travel distance was 19.6 km (95% CI 14.4;24.8) for patients with a pension compared with 35.1 km (95% CI 22.0;48.2) for patients without a pension, corresponding to a mean difference of 15.5 km (95% CI 1.3;29.7) in favor of patients with a pension.

**Fig. 2:**
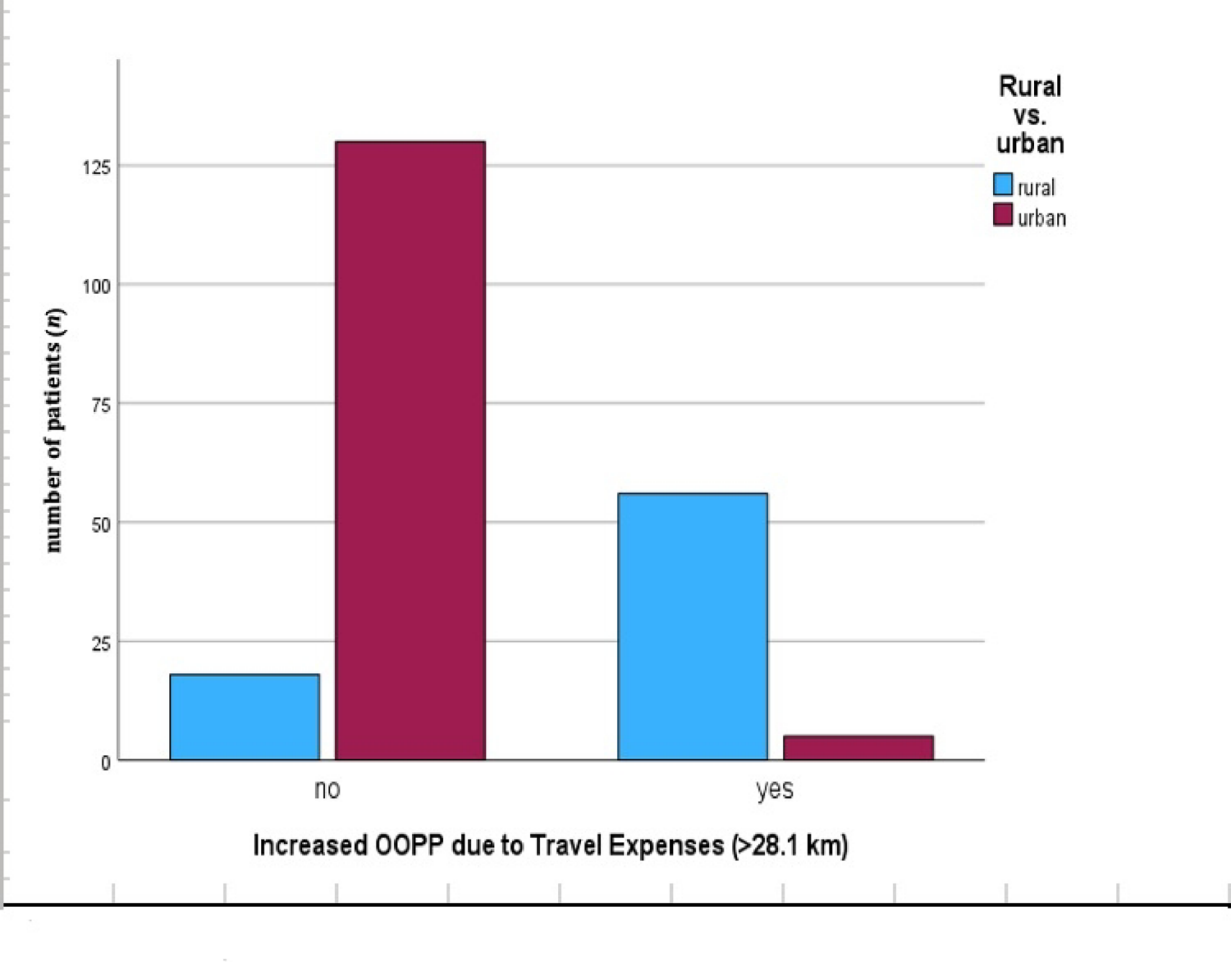
Association of reported OOPP in comparison to distance to the Cancer Care Center

Patients living in rural areas reported significantly increased OOPP due to travel expenses (Fig. 2). The sub-analysis of patients living between 10 and 28 km and over 28.1 km apart shows that if patients reported OOPP, living further away (over 28.1 km) was significantly more likely to be due to travel expenses than additional charges (compare Fig. 1b).

Patients residing in rural areas showed significantly higher scores on the financial difficulties scale of the EORTC QLQ-C30 compared to patients residing in urban areas (*p* < 0.001), as illustrated in Table 3. Patients residing outside of Leipzig (> 28 km away) also exhibited significantly elevated scores for the symptom scales "nausea and vomiting" (*p* = 0.043), "insomnia" (*p* = 0.024), and "appetite loss" (*p* = 0.015).

Among patients living within 28.1 km of Leipzig, the mean global health QoL score was lower (56.2; 95% CI 11.2;101.2) compared to those living outside Leipzig (70.8; 95% CI 20.5;121.2), corresponding to a moderate effect size (*Cohen’s d* = 0.62, 95% CI 0.0;1.2).

A similar pattern was observed when comparing patients with FB residing in Leipzig with those living more than 28.1 km away. The mean global health QoL scales were 56.2 (95% CI 11.2;101.2) and 68.6 (95% CI 26.8;110.5), respectively, with a moderate effect size (*Cohen’s d* = 0.55, 95% CI 0.0;1.1).

The sub-analysis of the global health QoL scale showed that patients residing in Leipzig without FB had in mean a higher score compared to patients residing in and outside Leipzig with reported FB. However, this trend did not reach statistical significance due to multiple testing and the limited number of participants within the six subgroups.

Without considering financial risk factors OOPP, financial difficulties, financial burden etc., the EORTC Q30 Global Health QoL Score was highest outside Leipzig < 28.1 km (OR 3.825, 95% CI 1.423;10.28) and only slightly higher > 28.1 km (OR 1.572, 95% CI 0.738;3.344) compared to the city of Leipzig.

In univariate analyses, a correlation between financial difficulties according to FD Score > 0 and Global QoL Score below median (OR 4.743, 95% CI 2.506;8.901, *p* = 7.77·10^–7^). This correlation was seen also in multivariate logistic regression. Here a low Global QoL Score below median again was predictive for financial difficulties and the absence of OOPP was protective for financial difficulties FD (OR _FD Score >0_ 0.211, 95% CI 0.098;0.451; *p* < 0.001) corresponding to a predicted four-fold risk of increased OOPP for financial difficulties according to FD score > 0 (OR 4.743, 95% CI 2.216;10.155, *p* < 0.001). In this model, a travel distance < 28.1 km was an independent risk factor for financial difficulties FD score > 0 (OR 3.054, 95% CI 1.033;9.032, *p* = 0.044). After excluding OOPP from the model, the travel distance < 28.1 km remained an independent risk factor for financial difficulties FD score > 0 (OR 3.231, 95% CI 1.155;9.036, *p* = 0.026).

Using the data obtained from the Microcensus 2021 we compared the mean net income of residents and HNC patients living in the same postcode area, as shown in Table 2. Net income was lower among patients with head and neck cancer compared with residents of the same postcode area (Δ − 499.56 €; 95% CI − 721.40; − 277.72 €; *p* = 0.014). The net income among HNC patients living in Leipzig (1196.75 €, 95% CI 1088.62;1304.88 €) was lower compared to HNC patients living outside of Leipzig < 28.1km (1854.53, 95% CI 1409.66;2299.40 €) and also lower than rural HNC patients > 28.1 km (1397.11 €, 95% CI 1120.93;1673.30 €).

**Table 2:** a-c: Overview of the net income, the individual delta and ratio per months in € before diagnosis of the cohort compared to residents living in the same ZIP code. Data on monthly mean net income per ZIP code were obtained from the 2021 results of the Micro census (largest annual household survey of official statistics in Germany) from Saxony. a Leipzig vs. Outside Leipzig > 28.1 km. b Outside Leipzig < 28.1 km vs. Outside Leipzig > 28.1 km. c Leipzig vs. Outside Leipzig < 28.1 km. Bold *p* values represent significant findings (p < 0.05)

Variables	Total valid	Mean (95% CI)		Cohen´s d	*p*-value
(*a*)					
Net income per months (€) patients before diagnosis				− 0.251 (− 0.567; 0.065)	0.1894
Leipzig	110	1196.75 (1088.62; 1304.88)	€		
Outside Leipzig > 28.1 km	60	1397.11 (1120.93; 1673.30)	€		
Net income per months (€) ZIP region				1.461 (1.047; 1.87)	**6.05·10**^**–25**^
Leipzig	111	1734.33 (1691.71; 1776.96)	€		
Outside Leipzig > 28.1 km	36	1442.33 (1434.72; 1449.95)	€		
Individual Delta in net income per months (€) before diagnosis vs. ZIP region				− 0.79 (− 1.268; − 0.307)	**0.0013**
Leipzig	61	− 499.56 (− 721.4; − 277.72)	€		
Outside Leipzig > 28.1 km	25	291.12 (− 197.38; 779.62)	€		
Ratio of net income per months (€) before diagnosis vs. ZIP region				− 0.877 (− 1.271; − 0.479)	**0.0022**
Leipzig	98	0.78 (0.72; 0.83)			
Outside Leipzig > 28.1 km	36	1.12 (0.92; 1.32)			
(*b*)					
Net income per months (€) patients before diagnosis				0.378 (− 0.036; 0.790)	0.0735
Outside Leipzig < 28.1 km	37	1854.53 (1409.66; 2299.40)	€		
Outside Leipzig > 28.1 km	60	1397.11 (1120.93; 1673.30)	€		
Net income per months (€) ZIP region				0.691 (0.189; 1.187)	**0.0068**
Outside Leipzig < 28.1 km	30	1457.33 (1450.32; 1464.35)	€		
Outside Leipzig > 28.1 km	36	1442.33 (1434.72; 1449.95)	€		
Individual Delta in net income per months (€) before diagnosis vs. ZIP region				0.060 (− 0.513; 0.633)	0.8384
Outside Leipzig < 28.1 km	22	372.09 (− 238.22; 982.40)	€		
Outside Leipzig > 28.1 km	25	291.12 (− 197.38; 779.62)	€		
Ratio of net income per months (€) before diagnosis vs. ZIP region				0.408 (− 0.103; 0.916)	0.1179
Outside Leipzig < 28.1 km	26	1.36 (1.16; 1.56)			
Outside Leipzig > 28.1 km	36	1.12 (0.92; 1.32)			
(*c*)					
Net income per months (€) patients before diagnosis				− 0.773 (− 1.154; − 0.388)	**0.0075**
Leipzig	110	1196.75 (1088.62; 1304.88)	€		
Outside Leipzig < 28.1 km	37	1854.53 (1409.66; 2299.40)	€		
Net income per months (€) ZIP region				1.358 (0.922; 1.789)	**2.27·10**^**–23**^
Leipzig	111	1734.33 (1691.71; 1776.96)	€		
Outside Leipzig < 28.1 km	30	1457.33 (1450.32; 1464.35)	€		
Individual Delta in net income per months (€) before diagnosis vs. ZIP region				− 0.819 (− 1.32; − 0.313)	**0.014**
Leipzig	61	− 499.56 (− 721.4; − 277.72)	€		
Outside Leipzig < 28.1 km	22	372.09 (− 238.22; 982.40)	€		
Ratio of net income per months (€) before diagnosis vs. ZIP region				− 1.705 (− 2.184; − 1.220)	**6.66·10**^**–6**^
Leipzig	98	0.78 (0.72; 0.83)			
Outside Leipzig < 28.1 km	26	1.36 (1.16; 1.56)			

## Discussion

The present study provides preliminary insights into the interplay between travel distance, financial burden, and quality of life among patients with head and neck cancer, a topic of growing relevance in the context of ongoing healthcare centralization.

Recent evidence indicates that a considerable proportion of HNC survivors experience a significant financial burden (FB), even within a statutory health insurance system (Rast et al. 2024). A notable finding of this study is that patients with HNC exhibited a lower net income compared to the general population within the same postal code areas. Several factors may contribute to this disparity. First, the disease itself is often associated with substantial income loss due to treatment-related morbidity and prolonged periods of work incapacity (Rast et al. 2024). In particular, HNC patients frequently experience functional impairments affecting speech, swallowing, and overall physical performance, which can limit their ability to return to work or maintain previous employment levels (Rosi-Schumacher et al. 2023; Zebralla et al. 2021). Second, higher rates of sick leave and early retirement in this patient group may further exacerbate financial constraints (Zebralla et al. 2018). Finally, pre-existing socioeconomic risk factors—such as lower educational attainment, higher prevalence of smoking, and employment in physically demanding or less secure occupations—may predispose individuals to both the development of HNC and a lower baseline income. Together, these aspects suggest that the observed income disparity is likely multifactorial and highlights the importance of considering socioeconomic vulnerability when evaluating FB and QoL in HNC survivors.

The role of travel distance (or, more precisely, travel burden) requires nuanced consideration. Although our analysis aimed to evaluate the impact of patients’ distance from the hospital on FB and living conditions, it is important to interpret the results in light of existing literature. For instance, a large, registry-based study of 1,921 German HNC patients at a tertiary center found no significant association between travel distance and UICC stage at diagnosis or survival outcomes. Our results are consistent with these findings, as we did not observe a significant association between travel distance and cTNM classification or UICC stage (Vahl et al. 2021).

The authors conclude that "travel burden is not synonymous with travel distance alone" and is influenced by infrastructural, sociodemographic, wealth and other system-level factors. Accordingly, while our data may show an association between residential distance and FB in terms of the impact by OOPP factors, the underlying mechanism is likely multifactorial. Transportation costs, time spent away from work, accommodation needs, rural/urban disparities, social structures at home and indirect productivity losses all contribute to the overall burden.

Our findings on the impact of travel distance on FB among German head and neck cancer survivors complement recent evidence on a previously published work by Pannenbecker et al. (2026). While their bicentric study highlights how treatment-related sequelae impair functional recovery and work reintegration, our results suggest that structural factors, such as the distance to treatment centers may further exacerbate these challenges through increased out-of-pocket costs, time investment, and productivity loss. The higher financial difficulty scores observed among patients from rural areas in the EORTC QLQ-C30 (Table 3) may reflect additional indirect costs associated with follow-up care, including longer travel distances, transportation expenses, and reduced accessibility of specialized healthcare services. This financial strain may indirectly affect QoL by limiting access to supportive care, increasing psychosocial stress, and delaying return to work. These challenges may be particularly pronounced among younger HNC patients residing in rural areas, who often face limited access to vocational rehabilitation and work integration programs (Miller et al. 2023).

**Table 3:** Quality of life comparison bewtween the subgroup urban (< 28 km) vs. rural (> 28 km) regarding the functioning scales and the symptom subscales/items of the EORTC-QLQ Q30 questionaire. Note: Kruskall–Wallis-Test for independent variables was performed. (a) null hypothesis: QoL functioning scale/symptom scale is between the subgroup Leipzig vs. Outside of Leipzig identical. Bold *p* values represent significant findings (*p* < 0.05)

EORTC-QLQ Q30 analysis urban < 28.1 km vs rural > 28.1 km		
	Null hypothesis (a)	*p*
Functioning scales		
Global health QoL	Fail to reject	0.132
Physical function	Fail to reject	0.488
Role function	Fail to reject	0.073
Emotional function	Fail to reject	0.058
Cognitive function	Fail to reject	0.177
Social function	Fail to reject	0.092
Symptom scales		
Fatigue	Fail to reject	0.052
Nausea and vomitting	**Reject**	**0.043**
Pain	Fail to reject	0.178
Dyspnoe	Fail to reject	0.640
Insomnia	**Reject**	**0.024**
Appetite loss	**Reject**	**0.015**
Constipation	Fail to reject	0.374
Diarrhoe	Fail to reject	0.196
Financial difficulties	**Reject**	** < 0.001**

Indeed, one main reason could still be the access to cancer care. According to an American Society of Clinical Oncology (ASCO) workforce analysis, just 3% of medical oncologists serve rural areas, even though 20% of Americans live there, and over 70% of U.S. counties have no medical oncologist (Charlton et al. 2015; Kirkwood et al. 2014). Many countries in Europe, including France, Scotland (Murchie et al. 2025), Italy (Sant et al. 2012), and Sweden (EU Country Cancer Profile Sweden 2025), have also reported disparities in access to cancer care. Not participating in dedicated organ-specific cancer-aftercare programs increases the risk of undetected relapse. This is especially the case as long as symptoms related to late toxicity and newly emerging signs of relapse(Böhm et al. 2022; Kawecki and Krajewski 2014) present in mixture and remain subclinical or difficult to diagnose by a general practitioner with limited experience in HNC diagnosis and access to diagnostic tools (Van De Weerd et al. 2026).

Nevertheless, initiatives to reduce disparities in rural cancer care are emerging. Cavanna et al. described a territorial oncology care program in Northern Italy that delivers treatment close to patients’ homes and achieved very high patient satisfaction (Cavanna et al. 2023)].

Our analysis revealed that there were differences in the reported QoL between patients residing in close proximity to the CCC and those residing at a greater distance. The distance and the associated OOPP due to travel burden and non-reimbursed payments are a cause for FB and therefore could influence the QoL. Recent research from our group showed that patients with FB have a significantly higher risk of deteriorated quality of life, with moderate effects and stronger associations for OOPP as a driver of FB compared with income loss (Rast et al. 2025).

Since the COVID-19 pandemic, telemedicine has been increasingly adopted and widely accepted by both oncological and non-oncological patients, offering a potential strategy to mitigate travel-related burden. Early evidence supports the feasibility and acceptability of telemedicine in HNC care. Van Rhee et al. demonstrated high patient satisfaction with decentralized telemedicine-based follow-up for premalignant and early-stage glottic carcinoma (Van Rhee et al. 2025).

The applicability of hybrid care models across all cancer types, especially HNC, is limited by requirements such as specialized examinations, imaging, and digital infrastructure. This emphasizes the need for disease-specific telemedicine approaches rather than universal solutions. While hybrid care can help reduce geographic barriers and the corresponding FB, further multicenter studies are needed to assess scalability, cost impact, quality of life, and patient suitability.

Upon examining the cancer characteristics, we also found contradictory findings. Monomodal surgery for cancer treatment often indicates an early stage of cancer with a small tumor size and/or the absence of locoregional metastasis. Such cases are typically associated with patients who experience early symptoms and seek medical attention quickly. This may explain the significant number of patients in Leipzig who were treated with surgery alone (*p* = 0.0417) and who live in an area with a high concentration of general practitioners and specialists, *e.g.*, otolaryngologists. But despite not reaching statistically significance, there are more patients with T3 or T4 or N3 (according to the 7th TNM 2017 edition) living in Leipzig compared to the groups outside of Leipzig (Table 1). These apparently contradictory findings point to a multifactorial underlying mechanism that requires more in-depth investigation. In addition to residence status, the social demographic parameters within a city must also be included in these analyses, e.g. by comparing high-privileged *versus* low-privileged neighborhoods based on postal codes. It is well known that patients with HNSCC tend to be less educated, poorer, and sicker with higher burden of comorbidities, and therefore are incapable to seek medical care (Massa et al. 2019).

Furthermore, patients with financial burden residing in Leipzig reported lower global health scales compared with patients living in rural areas. Notably, although a higher proportion of rural patients reported OOPP, they nevertheless demonstrated better global health scores. This finding suggests the presence of adaptive coping mechanisms that are not yet fully understood and highlights the complex, multifactorial relationship between financial burden, OOPP, and QoL. On average, working patients in our sample lived 15.5 km further away from the CCC. In terms of the calculated standard deviation of the distance between retired and working patients, the retired patients have a moderately higher average distance to the CCC. This could suggest that retired HNC survivors, due to logistical and financial challenges (greater OOPP due to travel), are reliant on living in urban areas when seeking medical services. Future research should account for OOPP in relation to net income to better assess their relative impact and clarify their relevance within the overall economic situation of patients with HNC.

Beyond travel-related challenges, financial burden remains a critical barrier to patient participation in cancer care and research. Notably, Deng et al. reported that nearly half of patients participating in clinical trials experienced financial hardship, underscoring the importance of targeted educational and financial support (Deng et al. 2023). For HNC survivors, reducing travel distance alone may be insufficient; comprehensive strategies addressing both direct and indirect costs are essential to ensure equitable access to care and research participation.

Many Western countries offer support through cancer advocacy organizations, while Germany provides both social security benefits (sick pay, rehabilitation, and unemployment assistance) and additional aid from foundations (German Cancer Aid and German Childhood Cancer Foundation). However, our findings indicate that patients are often insufficiently informed about these resources or that the available support does not adequately offset their financial burden.

However, our study has several limitations that should be considered when interpreting the findings and planning future research. First, measures of “distance” usually rely on postal codes or linear kilometers, which may not accurately capture the true travel burden (e.g., route characteristics, mode of transportation, travel time, travel costs, and patient mobility limitations). As Vahl et al. (2021) note, infrastructure, transportation options, and local geography may substantially mediate the impact of distance on healthcare utilization. Future research should therefore incorporate more comprehensive travel-related metrics, including travel time, cost, and transport mode, and examine how these factors interact with socioeconomic status and regional deprivation. Second, the study population was recruited exclusively from patients enrolled in an aftercare program at a single tertiary referral center. This may introduce selection bias, as patients who participate in structured follow-up programs at specialized centers may differ systematically from the broader HNC population in terms of health awareness, motivation, access to care, socioeconomic background, or disease severity. Consequently, the generalizability of our findings to patients treated in non-tertiary settings, rural regions, ambulatory care centers, or underrepresented socioeconomic groups may be limited. Existing German research has likewise largely focused on outpatient HNC aftercare programs at tertiary centers, while representation of rural settings, ambulatory care structures, and diverse socioeconomic strata remains insufficient. Third, the cross-sectional design of the present study limits the ability to infer causal relationships or assess temporal changes in healthcare access and follow-up behavior. Longitudinal studies are therefore needed to evaluate how geographic barriers, patient behavior, and healthcare utilization evolve over time and how these factors may influence long-term oncologic outcomes. Finally, intervention studies are sparse: while screening is advocated, research is needed to demonstrate that financial support or travel-assistance programs can improve outcomes (QoL, adherence, survival) in HNC survivors.

Overall, our findings suggest that travel distance contributes to the financial burden experienced by HNC patients, although this relationship is clearly multifactorial and not yet fully understood. The complex interplay between treatment advances, rising healthcare costs, travel demands, access to care, and quality of life highlights a critical need for further research. Future studies should provide critical insights into the impact of travel burden on financial outcomes and may inform ongoing healthcare policy debates in Germany. In parallel, the implementation of standardized screening tools for financial and travel-related burden, alongside the integration of financial counseling and social support into multidisciplinary care, appears essential. The primary challenge will be addressing patients' needs while preserving efficient clinical workflows in high-volume centers.

## Conclusion

A significant proportion of HNC survivors encounter logistical and financial difficulties in attending their aftercare appointments due to the considerable distances involved. Disparities in financial challenges faced by patients living at greater distances from treatment centers are linked to reduced QoL even in healthcare systems with statutory insurance and are of concern particularly in the context of cancer aftercare programs. It is therefore essential that routine screening for FB is integrated into oncological care, as early identification can guide interventions aimed at reducing its negative impact on treatment outcomes. Healthcare providers should proactively assess the situation of the patient and collaboratively plan follow-up care. If necessary, patients should be referred to specialized support services, such as social workers, psycho-oncologists, or patient navigators, to facilitate access to financial resources. The implementation of tailored support programs, encompassing financial counselling, has the potential to enhance the quality of life of this under-served population.

## Supplementary Information

Below is the link to the electronic supplementary material.

Supplementary file 1

## Data Availability

No datasets were generated or analysed during the current study.
