# Supplementary material for: The influence of urban versus rural residence and travel distance on net income, out-of-pocket payments and quality of life in German head and neck cancer survivors
Source: J Cancer Res Clin Oncol. 2026 Jul 18;152(7):144. doi: 10.1007/s00432-026-06534-5 (PMC13388607; doi:10.1007/s00432-026-06534-5)
Supplement: Supplementary file 1 [file 432_2026_6534_MOESM1_ESM.docx]

## Patient Questionnaire

“Economic Impact of Head and Neck Tumors and Their Treatment”

*(translated from the original version in German)*

## A) Sociodemographic Data

1. Gender
□ Male
□ Female

2. How old are you?
_____ years

3. What is your marital status?
□ Single
□ Married
□ Living separately
□ Divorced
□ Widowed
□ With partner
□ Other: __________

4. How many people currently live in your household?
□ I live alone
□ I live with my partner
□ I live with my spouse
□ I live with another adult: ________
□ I am a single parent
Minor children living in the household: ____ (number)

5. Your highest school qualification
□ No qualification
□ Secondary school certificate / elementary school
□ Intermediate secondary school certificate
□ Advanced technical college entrance qualification
□ General higher education entrance qualification
□ Other: ________

6. What vocational training do you have?
□ Apprenticeship
□ Technical school (master craftsman, technical college, vocational academy)
□ Advanced technical college qualification
□ University degree
□ No vocational training
□ Other vocational training: ________

7. Do you suffer from any other illnesses in addition to your cancer?
□ Yes, namely: ___________________________
□ No

8. What type of health insurance do you have?
□ Private
□ Statutory
□ Co-insured free of charge
□ Other, namely: __________________

## B) Employment and Income

9. What was your employment status at the time of tumor diagnosis?
□ Employed
□ Self-employed or freelance
□ Civil servant
□ Part-time employed
□ Unemployed
□ Retired

If you were already retired at the time of diagnosis, please continue with Section C – Question 18.

10. What was your net income before tumor diagnosis?
□ No personal income
□ Under €500
□ €501–1,000
□ €1,001–1,500
□ €1,501–2,000
□ €2,001–2,500
□ €2,501–3,000
□ €3,001–3,500
□ Over €3,500

11. What is your current employment status?
□ Employed
□ Self-employed or freelance
□ Civil servant
□ Part-time employed
□ Unemployed
□ Retired

a) If your employment status has changed since diagnosis, what is the reason?
□ Retirement due to age
□ Retirement due to tumor diagnosis
□ Career change due to tumor diagnosis
□ Career change for other reasons
□ Job loss due to tumor diagnosis
□ Job loss for other reasons
□ Reduction in working hours due to tumor diagnosis
□ Reduction in working hours for other reasons
□ Permanent sick leave due to tumor diagnosis
□ Permanent sick leave for other reasons
□ Other reason: __________

b) If you are currently employed, is your employment contract temporary or permanent?
□ Temporary employment contract
□ Permanent employment contract

c) If you are not employed, what is the reason?
□ I am looking for work
□ I cannot work due to illness
□ I do not wish to work
□ Other: __________________________

12. For how many months have you been on sick leave?
_____ months
□ I am not on sick leave

13. Do you currently have your own income?
□ Yes
□ No

b) If yes, what is your current monthly net income, i.e. the amount remaining after taxes and social insurance contributions?
□ Under €500
□ €501–1,000
□ €1,001–1,500
□ €1,501–2,000
□ €2,001–2,500
□ €2,501–3,000
□ €3,001–3,500
□ Over €3,500

14. Are other people financially dependent on your income?
□ Yes, namely: ____ additional persons
□ No, only myself

15. What is your current total available monthly net family income?
□ €501–1,000
□ €1,001–1,500
□ €1,501–2,000
□ €2,001–2,500
□ €2,501–3,000
□ €3,001–3,500
□ €3,501–4,000
□ €4,001–4,500
□ €4,501–5,000
□ Over €5,000

16. Have you suffered a loss of income due to your tumor disease?
□ Yes
□ No

a) If yes, how much per month?
□ Under €100
□ €100–200
□ €201–500
□ €501–800
□ €801–1,200
□ Over €1,200

b) If yes, do you have the possibility to compensate for these losses? (Multiple answers possible)
□ Yes, through loans
□ Yes, through savings or assets
□ Yes, through other means: __________________
□ No

17. Have you applied for a pension after tumor diagnosis?
□ Yes
□ No

a) If yes, how did this come about?
□ My health insurance provider requested it
□ My employer requested it
□ I submitted the application on my own initiative

## C) Expenses Since Tumor Diagnosis

18. Have you incurred higher expenses as a result of your tumor disease? (e.g. taxi costs, co-payments for medication, treatments)
□ Yes
□ No

a) If yes, how much per month?
□ Under €100
□ €100–200
□ €201–500
□ €501–800
□ €801–1,200
□ Over €1,200

b) If yes, what causes these higher expenses? (Multiple answers possible)
□ Co-payments
□ Travel expenses
□ Household assistance
□ Treatments and medications not reimbursed by health insurance
□ Other: __________________________

19. Has your tumor disease led to an overall deterioration in your living conditions?
□ Yes
□ No

a) If yes, where specifically do you have to cut back? (Multiple answers possible)
□ Leisure activities (e.g. cinema visits, holidays)
□ Food and nutrition
□ Housing/household expenses (e.g. rent, electricity, heating)
□ Medical treatments/additional services
□ Other: __________________________

20. Which of the following benefits are you currently receiving?
□ Wages/salary
□ Pension
□ Sick pay
□ Disability pension
□ Unemployment benefit I
□ Unemployment benefit II / Hartz IV
□ Basic income support
□ Social welfare
□ Occupational disability insurance benefits
□ Other, namely: __________________

21. Have / did you have difficulties obtaining the above-mentioned benefits?
□ Yes, because: __________________
□ No

22. Did you undergo rehabilitation after therapy?
□ Yes
□ No

a) If yes, how was it carried out?
□ Inpatient
□ Outpatient
□ Inpatient and outpatient

23. What were important goals of rehabilitation for you? (Multiple answers possible)
□ Recovery from therapy / rest
□ Professional reintegration
□ Receiving a pension
□ Meeting other affected persons
□ Physical fitness
□ Other: __________________

24. What was your relationship status at the time of rehabilitation?
□ Single
□ Married
□ Living separately
□ Divorced
□ Widowed
□ With partner
□ Other: __________________

## Current Health Status

We would like to find out how good or bad your health is TODAY.

This scale is numbered from 0 to 100.
100 represents the best health you can imagine.
0 represents the worst health you can imagine.

Please mark the point on the scale that best describes your health TODAY.

Now please enter the number you marked on the scale in the box below:

YOUR HEALTH TODAY = __________

Thank you very much for your participation!
